# Performance of SOI Bragg Grating Ring Resonator for Nonlinear Sensing Applications

**DOI:** 10.3390/s140916017

**Published:** 2014-08-28

**Authors:** Francesco De Leonardis, Carlo Edoardo Campanella, Benedetto Troia, Anna Gina Perri, Vittorio M. N. Passaro

**Affiliations:** 1 Photonics Research Group, Dipartimento di Ingegneria Elettrica e dell'Informazione, Politecnico di Bari, via E. Orabona n. 4, 70125 Bari, Italy; E-Mails: francesco.deleonardis@poliba.it (F.D.L.); edoardo.campanella81@gmail.com (C.E.C.); benedetto.troia@poliba.it (B.T.); 2 Laboratorio di Dispositivi Elettronici, Dipartimento di Ingegneria Elettrica e dell'Informazione, Politecnico di Bari, via E. Orabona n. 4, 70125 Bari, Italy; E-Mail: annagina.perri@poliba.it

**Keywords:** integrated optics, optical waveguides, nonlinear optics, sensors, coupled resonators

## Abstract

In this paper, a spectroscopic sensor formed by a silicon-on-insulator waveguiding Bragg grating ring resonator working in linear and non-linear regime is proposed. In linear regime, the device shows a spectral response characterized by a photonic band gap (PBG). Very close to the band gap edges, the resonant structure exhibits split modes having a splitting magnitude equal to the PBG spectral extension, whose characteristics can be exploited to obtain a RI optical sensor almost insensitive to the fabrication tolerances and environmental perturbations. When the device operates in nonlinear regime, exactly in the spectral region showing the split resonant modes, the RI sensing performance is strongly improved with respect to the linear regime. This improvement, demonstrated by taking into account all the non-linear effects excited in the integrated silicon structure (*i.e.*, Two Photon Absorption (TPA), TPA-induced Free Carrier Absorption, plasma dispersion, Self-Phase-Modulation and Cross-Phase-Modulation effects as induced by Kerr nonlinearity) as well as the deleterious thermal and stress effects, allows enhancing the performance of the RI split mode resonant sensors, while achieving good immunity to the fabrication tolerances and environmental perturbations. The improvement in terms of sensor resolution can be at least one order of magnitude, still without using optimal parameters.

## 1. Introduction

Optical ring resonators are fundamental devices for many Physics and Engineering application fields, ranging from quantum information [1], cavity optomechanics [2], telecommunications [3], to biochemistry [4–7]. Several configurations of optical ring resonators have been investigated and designed, whose modal and resonance spectral features strongly depend on geometrical parameters and physical effects involved. For example, the Fano resonances [4,5] and the Vernier effect [6] have been investigated in order to enhance the sensing performance of photonic sensors based on resonant microcavities. A detection limit enhancement in the nanoparticle (NP) sizing has been experimentally demonstrated by analyzing the split modes [8] arising from the presence of a NP along the perimeter of a Whispering Gallery Mode (WGM) cavity. Moreover, a self-referencing sensing scheme, based on the resonance splitting, has also been exploited to investigate the response of an optofluidic cavity system [9], where a common-mode noise suppression has been achieved by using two coupled ring resonators. Recently, the model and the experimental demonstration of an optical strain sensor formed by including a Fiber Bragg Grating (FBG) in a closed fiber loop has been carried out [10]. The spectral response shows a resonance splitting, associated with the degeneracy removal of two counter-propagating modes, which depend on FBG physical parameters. This splitting variation can therefore be used to sense the strain applied to the resonator in the region where the FBG is written, without suffering from the influence of fiber length variations due to any environmental perturbation. In addition, taking into account the increasing interest widely devoted to Silicon on Insulator (SOI) technology in the last decade [11], the development of photonic sensors based on this platform could induce a boost in the integration of electronic and photonic in one chip for ultra-high performance and very innovative sensing applications [12,13]. By considering together the advantages offered from the optical sensors exploiting split resonance mode structures [7–10] and those offered from SOI technology [11–13], we propose a SOI Bragg Microcavity Resonator (BMR) operating in linear and nonlinear regime to be used in RI sensing applications. Thus, the paper is organized as follows. In Section 2, we explain the physical operation of the device and briefly summarize the mathematical model to investigate the nonlinear effects occurring in the BMR device. The proposed modelling includes all nonlinear effects involved in the integrated silicon structure, *i.e.*, Two Photon Absorption (TPA), Free Carrier Absorption (FCA) induced by TPA, plasma dispersion effect, Self-Phase-Modulation (SPM) and Cross-Phase-Modulation (XPM) effects as induced by Kerr nonlinearity, and the grating coupling effects between clockwise and counter-clockwise waves. In addition, the model takes into account the mismatch between the input beam wavelengths and the microcavity resonance wavelengths, the coupling mechanism between the microcavity and input/output bus waveguide, and the temperature and stress effects on both optical guiding properties and nonlinear effective modal area characterizing the guiding structures. In Section 3, a number of numerical results are shown in order to evidence the physical features and the sensor operation. Moreover, a parametric investigation to find out the sensor design guidelines is carried out as well. Finally, Section 4 summarizes the conclusions.

## 2. Modeling of SOI Bragg Microcavity Resonator

**Figure 1.**
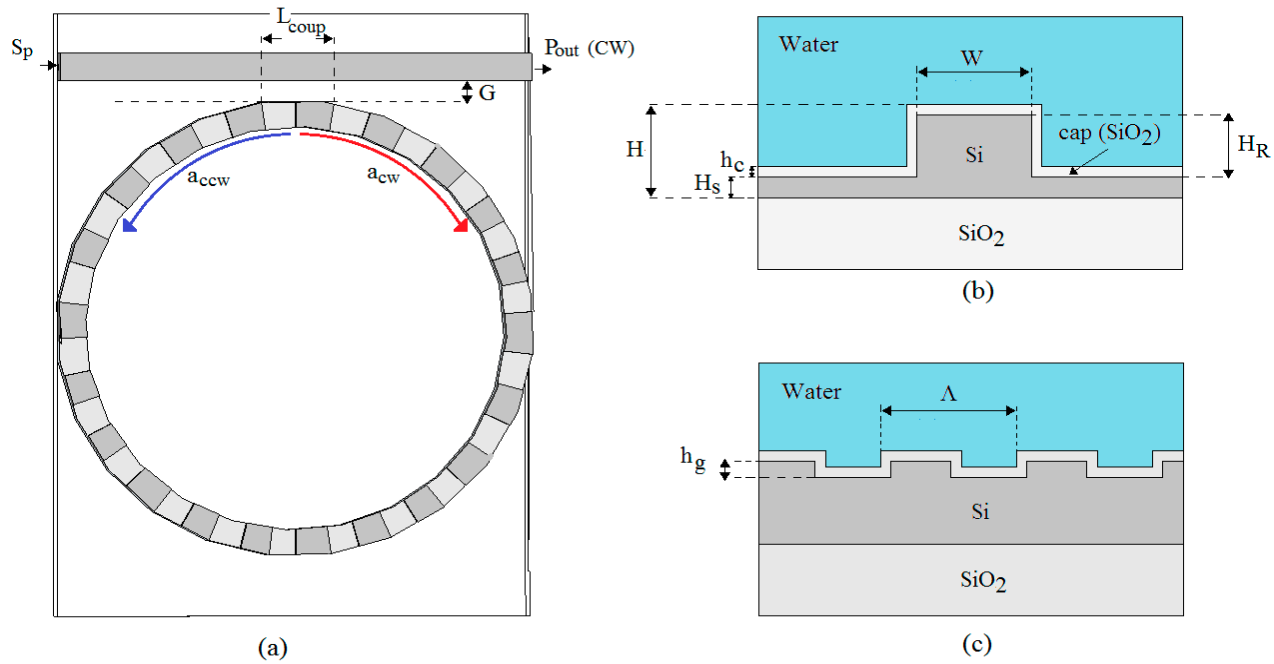
(**a**) Schematic architecture of Bragg microcavity resonator coupled to the external waveguides; (**b**) Silicon on Insulator (SOI) waveguide cross-section; (**c**) Bragg grating profile.

In this section, we briefly explain the nature of the splitting in a Bragg Microcavity Resonator (BMR) and propose the mathematical model to analyse the features occurring in a SOI BMR operating in linear and nonlinear regimes. The BMR geometry in sketched in Figure 1a, where a single bus waveguide is used to couple the input beam (*S_p_*) into the ring resonator having a cavity length (*L_cavity_*) equal to an integer number of the grating period (Λ). The grating depth is *h_g_*, as shown in Figure 1c.

**Figure 2.**
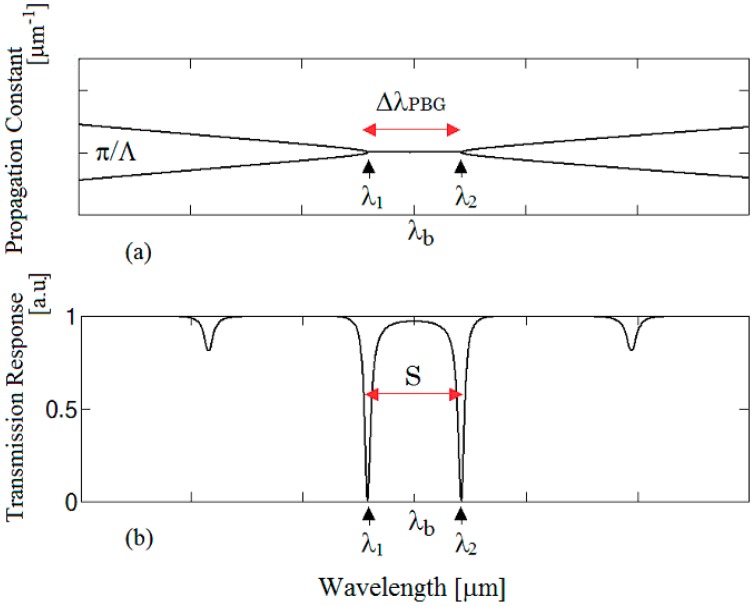
(**a**) Photonic Band Gap (PBG) spectral extension depending on *λ_1_* and *λ*_2_; (**b**) Qualitative transmission response in the spectral region around *λ_b_*.

Before describing the general mathematical model to include the nonlinear effects, it is needed to outline the basics of the BMR device. Its physical behavior and the theoretical model in linear regime have been experimentally proven in case of a Bragg grating included in a fiber ring resonator [10]. In fact, if the input wave (*S_p_*) has a wavelength close to Bragg wavelength (*λ_b_* = 2*n_eff_Λ*), two counter-propagating beams, coupled to each other, are originated inside the optical microcavity. This coupling, in its turn, induces two split resonances at *λ_1_* and *λ_2_* as in Figure 2b. In addition, the splitting magnitude can be directly evaluated as:

(1) $$\begin{array}{ll} \lambda_{1}=\frac{2n_{\text{eff}}\lambda_{b}}{2n_{\text{eff}}\left|\Delta n\right|}; & \lambda_{2}=\frac{2n_{\text{eff}}\lambda_{b}}{2n_{\text{eff}}-\left|\Delta n\right|} \end{array}$$

where *n_eff_* is the effective refractive index of the waveguide without grating, and Δ*n* is the index modulation depth. The dips composing the doublet (*i.e.*, *λ_1_* and *λ_2_*), generated near *λ_b_*, correspond to well separated “symmetric” and “anti-symmetric” resonant wavelengths (*i.e.*, Lorentzian profile modes) which lie at the Photonic Band Gap (PBG) edges. Thus, the amplitude of this splitting is exactly equal to Δ*λ_PBG_* (*i.e.*, the PBG extension), resulting in [10]:

(2) $$\Delta \lambda_{\text{PBG}}=\lambda_{1}-\lambda_{2}=\left(\frac{4n_{\text{eff}}\left|\Delta n\right|}{4{n_{\text{eff}}}^{2}-{\left|\Delta n\right|}^{2}}\right)\lambda_{b}$$

**Figure 3.**
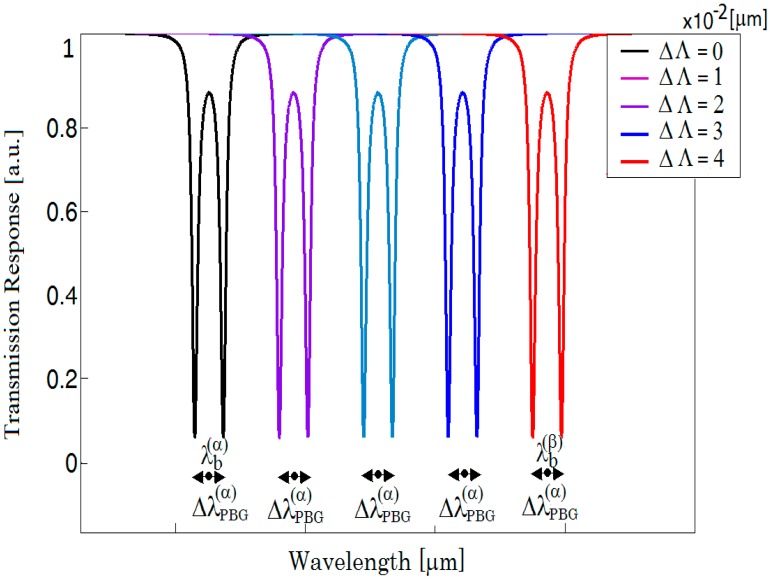
Bragg microcavity resonator transmission when *Λ* changes with Δ*Λ* = 1 × 10^−2^ μm.

To clarify this mode splitting, we report the eigenvalues of Bragg grating in Figure 2a and the BMR spectral response in Figure 2b. As evidenced by Equations (1) and (2), if the Bragg grating period varies, both the cavity length and the Bragg wavelength will change, inducing a shift of the split dips. However, their distance still remains constant because the PBG extension remains almost unvaried. In fact, as reported in Figure 3, if *Λ* is changed to 1 × 10^−2^ μm, *λ_b_* passes from *λ_b_*^(^*^α^*^)^ to *λ_b_*^(^*^β^*^)^ and the splitting Δ*λ_PBG_^(α)^* keeps a constant magnitude.

This effect means that the device becomes almost insensitive to the length variations, associated with fabrication tolerances as experimentally proven in [14], but also to the environmental perturbations [10]. Once the splitting nature has been explained by focusing also on the advantage in terms of fabrication tolerances and insensitivity to environmental perturbations, we analyze the operation of the device when the non-linear effects are excited to investigate if potential performance improvements can be achieved. To this aim, a model based on a set of partial differential equations for the nonlinear coupling between clockwise (CW) and counter-clockwise (CCW) waves inside the BMR has been developed, including one rate equation for the hole-electron pairs generated in the resonator by the TPA effect as induced by the propagating waves.

The equations have been derived assuming the input pump (*S_p_*) as injected in the BMR by means of the evanescent coupling between the resonant microcavity and the input external bus, both based on rib waveguides in SOI technology platform. *G* and *L_coup_* are the directional coupler gap and length, respectively. Although the microcavity in Figure 1a is a ring resonator, the following model can be applied to any microcavity shape, such as race-track, ring or disk resonators. The cross-section of the not-etched section waveguide is sketched in Figure 1b, showing the main geometrical parameters such as the rib etch depth *H_R_* and width *W*, the overall waveguide height *H*, and the slab height *H_S_*. In addition, the optical device can be covered by water solution, as in Figure 1b. Finally, a SiO_2_ protective passivation layer has been also included in the waveguide cross section.

Without any lack of generality, we assume that the electric field inside the microcavity is predominantly a single transverse mode (single-mode condition). Thus, up to two propagating modes are assumed in the resonator, one quasi-TE (dominant horizontal *x*-component of electric field) and one quasi-TM (dominant vertical *y*-component), where *x* and *y* designate the waveguide cross section coordinates. Hereinafter, the input pump wave (*S_p_*) is aligned with quasi-TE or quasi-TM polarization. Thus, according to the full-vectorial nonlinear coupled mode theory [15], the equations describing the power transfer among the CW and CCW waves can be respectively written as:

(3) $$\begin{array}{l} \frac{\partial a_{cw}}{\partial t}+v_{g}\left(T,\sigma \right)\frac{\partial a_{cw}}{\partial z}=j\left[\omega_{0}\left(1-\frac{{\left|a_{cw}\right|}^{2}}{a_{0}^{2}}\right)-\omega \right]a_{cw}-\frac{1}{2}\frac{1}{\tau \left(T,\sigma \right)}a_{cw}-\frac{1}{2}v_{g}\left(T,\sigma \right)\alpha_{s}^{\left(\text{FCA}\right)}\left(T,\sigma \right)a_{cw}\\ -v_{g}\left(T,\sigma \right)\left[\frac{1}{2}\frac{\beta_{cw,\text{CCW}}^{\text{TPA}}\left(T,\sigma \right)}{A_{cw,cw}^{\text{TPA}}\left(T,\sigma \right)}{\left|a_{cw}\right|}^{2}+\frac{\beta_{cw,\text{CCW}}^{\text{TPA}}\left(T,\sigma \right)}{A_{cw,\text{CCW}}^{\text{TPA}}\left(T,\sigma \right)}{\left|a_{\text{CCW}}\right|}^{2}\right]a_{cw}+\\ +jv_{g}\left(T,\sigma \right)\left[\frac{\gamma_{s}\left(T,\sigma \right)}{A_{cw,cw}^{\text{Kerr}}\left(T,\sigma \right)}{\left|a_{cw}\right|}^{2}+2\frac{\gamma_{s}\left(T,\sigma \right)}{A_{cw,\text{CCW}}^{\text{Kerr}}\left(T,\sigma \right)}{\left|a_{\text{CCW}}\right|}^{2}\right]a_{cw}+\\ +jv_{g}\left(T,\sigma \right)\frac{2\pi }{\lambda }\Delta n\left(T,\sigma \right)a_{cw}-j\mu \left(T,\sigma \right)a_{\text{CCW}}e^{j2\delta z}-j\mu_{\text{Kerr}}\left(T,\sigma \right)a_{\text{CCW}}e^{j2\delta z}+\xi_{p}S_{p} \end{array}$$

(4) $$\begin{array}{l} \frac{\partial a_{\text{CCW}}}{\partial t}+v_{g}\left(T,\sigma \right)\frac{\partial a_{\text{CCW}}}{\partial z}=j\left[\omega_{0}\left(1-\frac{{\left|a_{\text{ccw}}\right|}^{2}}{a_{0}^{2}}\right)-\omega \right]a_{\text{CCW}}-\frac{1}{2}\frac{1}{\tau \left(T,\sigma \right)}a_{\text{CCW}}-\frac{1}{2}v_{g}\left(T,\sigma \right)\alpha_{s}^{\left(\text{FCA}\right)}\left(T,\sigma \right)a_{\text{CCW}}+\\ -v_{g}\left(T,\sigma \right)\left[\frac{1}{2}\frac{\beta_{\text{CCW},\text{CCW}}^{\text{TPA}}\left(T,\sigma \right)}{A_{\text{CCW},cw}^{\text{TPA}}\left(T,\sigma \right)}{\left|a_{\text{CCW}}\right|}^{2}+\frac{\beta_{\text{CCW},cw}^{\text{TPA}}\left(T,\sigma \right)}{A_{\text{CCW},cw}^{\text{TPA}}\left(T,\sigma \right)}{\left|a_{cw}\right|}^{2}\right]a_{\text{CCW}}+\\ +jv_{g}\left(T,\sigma \right)\left[\frac{\gamma \left(T,\sigma \right)}{A_{\text{CCW},\text{CCW}}^{\text{Kerr}}\left(T,\sigma \right)}{\left|a_{\text{CCW}}\right|}^{2}+2\frac{\gamma \left(T,\sigma \right)}{A_{\text{CCW},cw}^{\text{Kerr}}\left(T,\sigma \right)}{\left|a_{cw}\right|}^{2}\right]a_{\text{CCW}}+\\ +jv_{g}\left(T,\sigma \right)\frac{2\pi }{\lambda }\Delta n\left(T,\sigma \right)a_{\text{CCW}}-j\mu \left(T,\sigma \right)a_{cw}e^{-j2\delta z}-j\mu_{\text{Kerr}}\left(T,\sigma \right)a_{cw}e^{-j2\delta z} \end{array}$$

where *a_cw_*, *a_ccw_* represent the slowly varying field amplitudes (functions of the time (*t*), and the space (*z*) along the propagation direction) for the waves counter-propagating inside the Bragg microcavity resonator, being *v_g_* their group velocity. We consider the influence of the nonlinear effects such as TPA and Kerr effects on the features of the devices based on Bragg gratings integrated inside the resonant microcavities, by analysing that spectral region showing the split resonant modes. In addition, we generalize the model evaluating all optical parameters depending on temperature (*T*) and waveguide mechanical stress (*σ*), as it will be specified in the following. In the general formalism based on the full vectorial coupled mode theory [15], the terms *ω_0_[(1* − |*a_cw_*|*^2^/a_0_^2^)* − *ω]* and *ω_0_ [(1* − |*a_ccw_*|*^2^/a_0_^2^)* − *ω]*, indicate the mismatch from the resonance condition of CW and CCW waves, respectively. In particular, *ω* is the angular frequency of the input wave and *ω_0_* represents the resonant angular frequency of the microcavity without Bragg grating. Finally, the terms *(1* −|*a_cw_*|*^2^/a_0_^2^)* and *[(1−*|*a_ccw_*|*^2^/a_0_^2^)* indicate the shift of the resonances as induced by Kerr effect for CW and CCW wave, respectively, being *a_0_^2^* = *A_i_^TPA^(T,σ)n_0,Si_(T)/n_2_(T)*, with *n_0,Si_(T)* and *n_2_(T)*, the silicon linear and nonlinear refractive indexes, respectively. Similarly, the term *μ(T,σ)* represents the coupling coefficient between the CW and CCW waves induced by the Bragg grating. Moreover, the coefficient *μ_Kerr_(T,σ)* indicates the deviation from *μ(T,σ)* as induced by the presence of the Kerr effect. They are defined as follows:

(5a) $$\mu \left(T,\sigma \right)=\frac{v_{g}\pi }{2P_{N}Z_{0}\lambda }{\left(n_{0,Si}^{2}\left(T\right)-n_{\text{clad}}^{2}\right)}^{2}\frac{\sin\left(\pi Dc\right)}{\pi }\iint_{\text{grating}}{\left|E\left(x,y,T,\sigma \right)\right|}^{2}\text{dxdy}$$

(5b) $${\mu_{\text{Kerr}}\left(T,\sigma \right)=\frac{v_{g}\pi }{2P_{N}Z_{0}\lambda }{\left(n_{2}^{2}\left(T\right){\left(\frac{{\left|a_{i}\right|}^{2}}{A}\right)}^{2}+2n_{2}\left(T\right)n_{0,Si}\left(\frac{{\left|a_{i}\right|}^{2}}{A}\right)\right)}^{2}\frac{\sin\left(\pi Dc\right)}{\pi }\iint_{\text{grating}}{\left|E\left(x,y,T,\sigma \right)\right|}^{2}\text{dxdy}|}_{\text{i}\;=\;\text{CW},\text{CCW}}$$

where *Z_0_* is the free space impedance, *Dc* is the grating duty cycle, *E(x,y,T,σ)* represents the electric field of the unperturbed waveguide, and *P_N_* = *(1/4)∬0.5(**E*** × ***H**)* · **z̄** is the normalization coefficient. The term *τ* represents the overall photon decay time of the wave (CW or CCW) inside the BMR. It is related to the overall resonator quality factor by means of the relationship *Q* = *ωτ*, as:

(6) $$\frac{1}{\tau }=\frac{1}{\tau^{l}}+\frac{1}{\tau^{c}}$$

where the two contributions related to loss (*τ^l^*) and input bus coupling (*τ^c^*) time are constant. Furthermore, the decay time related to losses can be also given as a function of the overall linear loss coefficient (*α_loss_*) [15]. Moreover, the coefficient *ξ_p_* in Equation (3) is related to the power fraction transferred into the resonator from the input pump (*S_p_*) as: *ξ_p_* = *(v_g_/(τ^c^L_cavity_))^1/2^*, being *L_cavity_* the cavity length and *δ* the phase mismatch. Hereinafter, it is convenient to introduce the coupling factor *κ^2^(T,σ)*, defined as the power fraction of the input pump wave coupled between the BMR resonator and the external bus waveguide. It is possible to demonstrate that the coupling factor *κ^2^(T,σ)* is related to the coupling time constant *τ^c^* by means of the following equation [15]:

(7) $$\kappa^{2}=\frac{1}{\tau^{c}}\frac{L_{\text{cavity}}}{v_{g}}$$

Furthermore, in Equations (3) and (4), the terms *β_i,ψ_^TPA^(T)* (*I* = *CW,CCW*) represent the TPA effect on the *i^th^* beam induced by the *ψ^th^* wave (*ψ* = *CW,CCW*). Moreover, the coefficient *γ(T)* = *n_2_(T)ω/c* takes into account the SPM and XPM effects as induced by Kerr nonlinearity. Finally, we have also included in the model the thermal dependence for the coefficients *β_i,ψ_^TPA^(T)*, and *n_2_(T)* by considering the equations proposed in [16], thus realising a fully self-consistent approach. The effective modal area relevant to the *i^th^* wave (*i* = *CW,CCW*) plays a fundamental role since it determines the efficiency with which any nonlinear effect manifests inside the optical SOI waveguide. According to the full-vectorial coupled mode theory [15], the effective modal areas are calculated as:

(8) $${A_{i,\psi }^{\text{TPA}}\left(T,\sigma \right)=A_{i,\psi }^{\text{Kerr}}\left(T,\sigma \right)=\frac{4\mu_{0}\left({\hat{N}}_{i}{\hat{N}}_{\psi }\right)}{{ɛ}_{0}n_{0,Si}}{\left[\frac{1}{3}\iint \left({\left|{\text{e}}_{i}\right|}^{2}{\left|{\text{e}}_{\psi }\right|}^{2}+{\left|{\text{e}}_{i}\cdot {\left({\text{e}}_{\psi }\right)}^{*}\right|}^{2}+{\left|{\text{e}}_{i}\cdot {\text{e}}_{\psi }\right|}^{2}\right)\text{dxdy}\right]}^{-1}|}_{i,\psi =CW,\text{CCW}}$$

where *n_0,Si_* is the silicon refractive index calculated through the generalized Sellmeier equation [17] at the pump wavelength and temperature *T*, and *N̂_i_* = *(1/4)∬0.5(**E*** × ***H**)*⋅ *z̄ dxdy*. In addition, in Equations (3) and (4) both FCA as induced by the change of free carrier density generated by TPA of CW and CCW waves (*α_i_^(FCA)^*), and effective index change due to plasma dispersion effect as induced by free carriers, have been evaluated [16]. Finally, the full physical consistence of the system (3)–(4) also requires the rate equation governing the free carrier dynamics into the waveguide core [15]:

(9) $${\frac{dN_{c}}{d\tau }=-\frac{N_{c}}{\tau_{\text{eff}}}+\frac{1}{2}\sum_{i}\frac{\beta_{i,i}^{\left(\text{TPA}\right)}\left(P_{i}^{2}\right)}{\hbar \omega {\left(A_{i,i}^{\left(\text{TPA}\right)}\right)}^{2}}+\frac{1}{2}\sum_{i}\sum_{\psi \neq i}\left(\frac{\beta_{\psi ,i}^{\text{TPA}}}{\hbar \omega \left(A_{\psi ,i}^{\text{TPA}}\right)\left(A_{\psi ,\psi }^{\text{TPA}}\right)}P_{i}P_{\psi }+\frac{\beta_{\psi ,i}^{\text{TPA}}}{\hbar \omega \left(A_{\psi ,i}^{\text{TPA}}\right)\left(A_{i,i}^{\left(\text{TPA}\right)}\right)}P_{i}P_{\psi }\right)|}_{i,\psi =CW,\text{CCW}}$$

where *τ_eff_* is the effective recombination lifetime for free carriers, *P_i_* is the optical power relevant to *i^th^* wave, and *h* is the reduced Planck constant.

As mentioned before, the proposed model includes the temperature and waveguide mechanical stress parameters in order to evaluate thermal and stress influence on the sensor performance. Thus, generally speaking, a thermal difference with respect to a reference temperature (*T_ref_*) induces strain and stress fields inside the optical waveguide, which are related as [18]:

(10) $$\left[\begin{array}{c} \sigma_{x}\\ \sigma_{y}\\ \sigma_{z} \end{array}\right]=\frac{E_{Y}}{\left(1+\nu \right)\left(1-2\nu \right)}\left[\begin{array}{ccc} 1-\nu & \nu & \nu \\ \nu & 1-\nu & \nu \\ \nu & \nu & 1-\nu \end{array}\right]\;\left[\begin{array}{c} {ɛ}_{x}\\ {ɛ}_{y}\\ {ɛ}_{z} \end{array}\right]-\frac{\alpha_{\text{therm}}E_{Y}\left(T-T_{\text{ref}}\right)}{\left(1-2\nu \right)}$$

where *σ_x,y,z_* and *ε_x,y,z_* represent the stress and strain component along *x*, *y*, and *z* direction, and *E_Y_*, *v* and *α_therm_* are the Young's modulus, the Poisson's ratio, and the thermal coefficient, respectively. In particular, the stress produces changes in material refractive index and, then, in the optical field distributions, influencing the effective index of the optical mode propagating in the guided-wave structure, the modal effective area values, and the grating Bragg coefficients. In fact, the stress-induced change in the material refractive index can be described by the following relations [18]:

(11) $$\{\begin{array}{c} n_{x}-n_{0}=-B_{1}\sigma_{x}-B_{2}\left(\sigma_{y}+\sigma_{z}\right)\\ n_{y}-n_{0}=-B_{1}\sigma_{y}-B_{2}\left(\sigma_{x}+\sigma_{z}\right) \end{array}$$

being *n*_0_ the refractive index of the material without stress (Si or SiO_2_), *B_1_* and *B_2_* the stress-optical constants dependent on the material photoelastic tensor (*p_ij_*) [18].

## 3. Results and Discussion

### 3.1. Physical Analysis

In this sub-section, a number of parametric simulations are proposed in order to find the physical features of BMR operating in linear and nonlinear regimes. In this context, it is worth describing briefly the procedure for the spectra calculations based on the model presented in the previous section. With the aim to realize self-consistent simulations, we have implemented an integrated algorithmic procedure based on both hand-made code and commercial software based on full-vectorial finite-element method (FEM) [19]. Consequently, we determine firstly the stress field in the waveguide for a fixed temperature value by solving numerically Equation (10) by FEM. It solves the static equilibrium equation, simultaneously satisfying the stress–strain relation, the thermal effects and the strain–displacement relation, with proper boundary conditions for the displacement variables in the *x*, *y*, and *z* directions. Once the stress distribution in the vicinity of the ridge waveguide has been calculated, the local refractive index distribution can be evaluated using Equation (11), and then FEM is used to solve the Maxwell's equations and determine the optical mode distributions and effective refractive index for both quasi-TE and quasi-TM polarizations at the operative wavelengths. It is worth outlining that the FEM electromagnetic module used in this step works together with the FEM stress module in order to take into account the stress effect on the material refractive index. Now, the electric-field distributions are used to calculate the grating coupling coefficient and the nonlinear effective modal area using Equations (5) and (8), respectively. Finally the hand-made code, solving the equations system (3), (4) and (9), allows the spectra for CW and CCW waves to be accurately evaluated.

In conclusion, the procedure described above gives the BMR features in both linear and nonlinear regimes, following an approach simultaneously integrated and multi-physics. Hereinafter, the simulations have been performed by setting typical physical parameters and waveguide geometry for silicon-based technology, as listed in Tables 1 and 2. According to Figure 1a, the transmittivity is defined as:

(12) $$T_{R}=\frac{P_{\text{out}}}{P_{i}}={\left|\frac{S_{p}-\kappa a_{cw}\left(\lambda \right)}{S_{p}}\right|}^{2}$$

**Figure 4.**
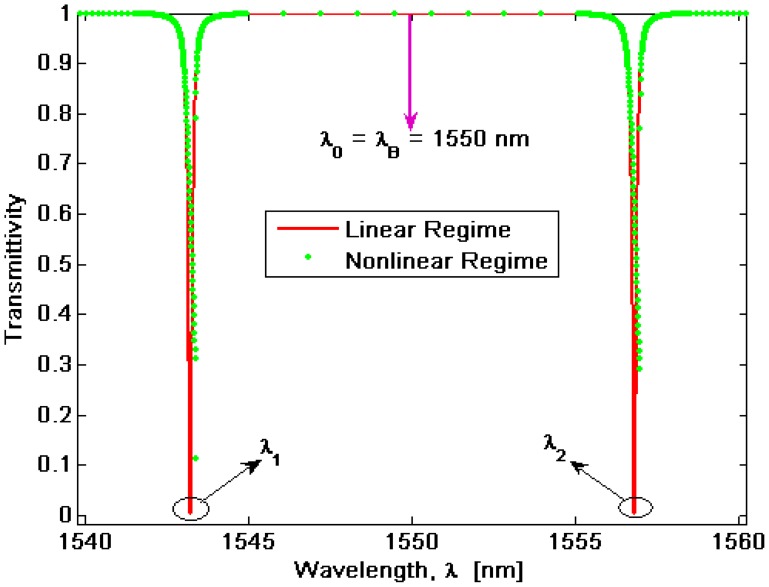
BMR spectral response in linear and nonlinear regime.

In Figure 4 the transmittivity is shown as a function of different values of input power. In the simulations, we have assumed a grating period of *Λ* ≅ *245.4* nm in order to satisfy the Bragg condition at *λ_B_* = 1550 nm, a cavity length *L_cavity_* = 92.28 μm corresponding to a period number *N_period_* = 376, *σ_i_* = −100 MPa, and *T* = *T_ref_* = 293 K.

About the initial stress *σ_i_*, we have introduced the external parameter *σ_i_*, defined as the in-plane stress component present in the uniform SiO_2_ upper cap film, far away from the ridge. In this work, the stress in the protective oxide layer induced by fabrication processes has been considered as well. Alternatively, the stress in the cap material can be altered by thermal anneals, typically assuming values ranging from −400 MPa to −100 MPa. The plot in Figure 4 clearly shows the resonance splitting effect, previously described in Section 2. The grating presence induces a coupling effect between CW and CCW waves, resulting in a split doublet with resonances placed at *λ_1_* = 1543.23 nm and *λ_2_* = 1556.82 nm, respectively.

In particular, for input powers lower than 2 mW (linear regime), both wavelengths *λ_1_* and *λ_2_* exclusively depend on the coefficient *μ(T,σ)* as well as on the decay time, according to:

(13a) $$\omega_{1,2}=\omega_{0}\pm \sqrt{\mu \left(T,\sigma \right)\sqrt{\left(\mu^{2}\left(T,\sigma \right)+4{\left(\frac{1}{2\tau }\right)}^{2}\right)}-{\left(\frac{1}{2\tau }\right)}^{2}}\;\text{CW}\;\text{wave}$$

(13b) $$\omega_{1,2}=\omega_{0}\pm \sqrt{\mu^{2}\left(T,\sigma \right)+{\left(\frac{1}{2\tau }\right)}^{2}}\text{CW wave}$$

**Figure 5.**
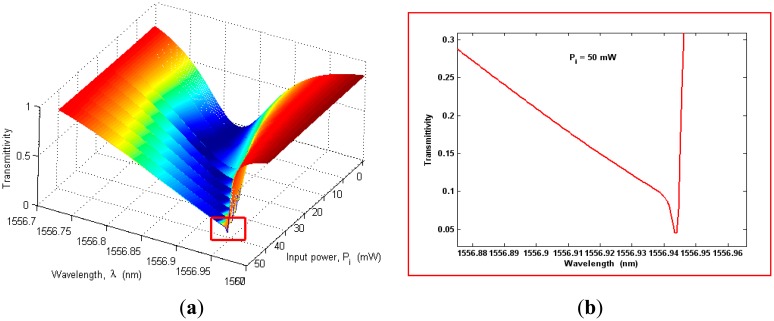
(**a**) Transmittivity in the spectral range around *λ_2_* as a function of the input power *P_i_*; (**b**) Zoom for *P_i_* = 50 mW.

Moreover, if the grating coupling coefficient is much larger than the photon decay rate, Equation (13) can be approximated by means of the relationship *ω_1,2_* ≅ *ω_0_* ± *μ(T,σ)*, but only in linear regime. In fact, as shown in Figure 4, by increasing the input power the transmittivity exhibits both a shift towards higher wavelengths and a spread of the resonance shape as induced by nonlinear effects. This is evidenced in Figure 5, where a zoom plot is shown around *λ_2_* as in Figure 5b. Therefore, Figure 5a,b clearly indicates that the nonlinear effects act by deforming the spectrum shape with respect to the Lorentzian profile, typical of the linear regime. Moreover, the degree of the deformation increases by increasing the input power. In addition, a very specific feature can be also observed for input powers larger than a threshold value, hereinafter indicated as *P̅_ι_*, depending on the microcavity parameters (in particular, on the cavity quality factor).

In fact, for *P_i_* ≥ *P̅_ι_* the transmittivity spectrum, even if totally deformed, presents a very narrow, shaped and deep spike, as evidenced in Figure 5b. This spike can be very useful to improve the sensor performance with respect to the linear regime, as it will be demonstrated in the next section.

**Figure 6.**
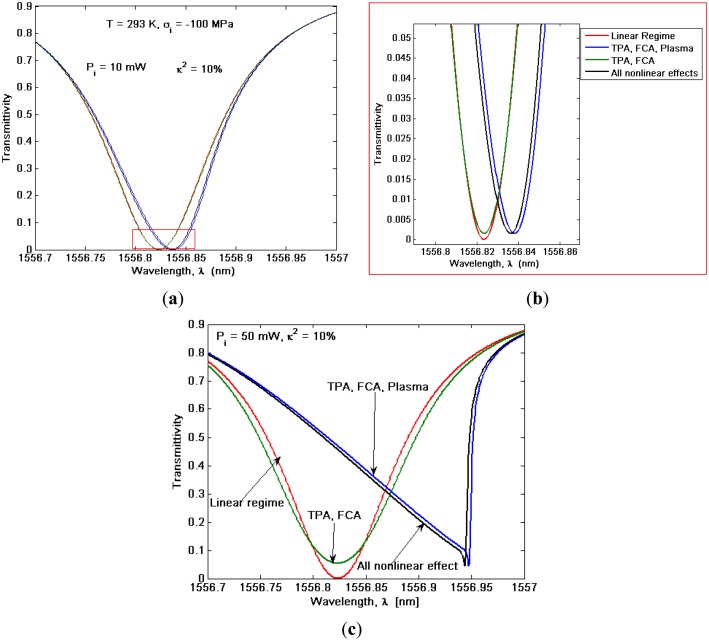
Transmittivity *versus* wavelength for different nonlinear effects: (**a**) input power *P_i_* = *10 mW* with (**b**) zoom plot; (**c**) input power *P_i_* = *50 mW*.

However, at this step, it is worth finding the physical reasons for the spike formation and we can refer to Figure 6a,b, where the transmittivity spectrum is shown in presence of nonlinear effects acting separately for *P_i_* = *10mW* ≤ *P̄_ι_* and *P_i_* = *50 mW* ≥ *P̄_ι_*, respectively. In this context, Figure 6 shows how the only presence of both TPA effect with induced FCA and plasma dispersion effects (blue curve) induce similar spectrum features with respect to the real case (black curve), but with a shift in the spike position. This offset is induced by the absence of the shift effect towards shorter wavelengths, depending on the Kerr effect. Moreover, by neglecting the TPA-induced plasma dispersion effect (see green curve), the transmittivity evidences only a spread with respect to the linear regime (red curve), without any wavelength shift. Therefore, with respect to the linear regime the resonance wavelength shift is determined by the competition between opposite trends as induced by Kerr and plasma dispersion effect, with a strength depending on the input power, as sketched in Figure 6b,c. Finally, we conclude that the spike formation can manifest only under the condition *P_i_* ≥ *P̄_ι_*, where a strong TPA effect, a not negligible spectrum red shift as induced by the high plasma dispersion effect as well as a higher FCA for wavelengths shorter than the spike wavelength, simultaneously occur and can be observed.

**Figure 7.**
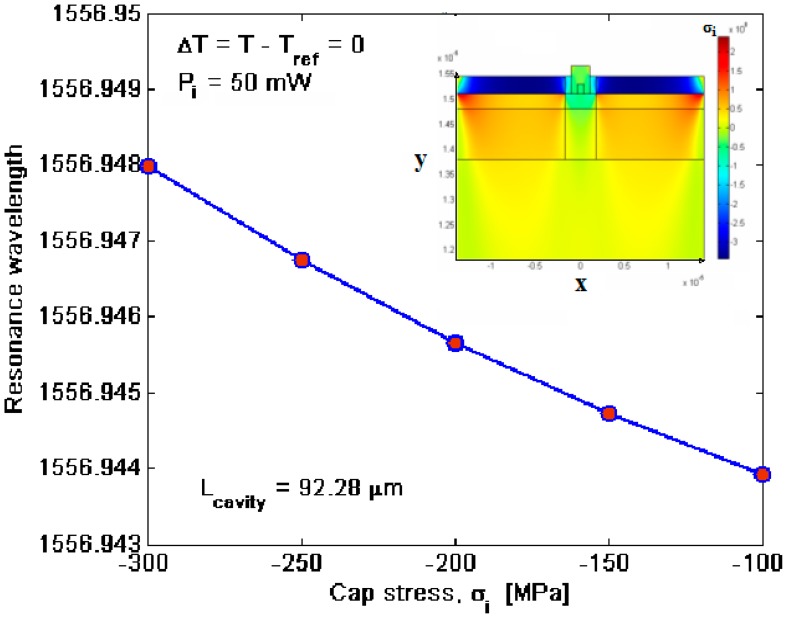
Resonance wavelength shift *versus* the cap layer stress; (inset) structure stress induced by *σ_i_* = −*400 Mpa*.

In addition, for *P_i_* < *P̄_ι_* the nonlinear effects only determine an increasing of optical losses due to TPA and FCA, being the plasma dispersion effect too weak to induce a sufficient red shift and, then, to produce the spike formation. In the following, we propose a number of parametric simulations to find both the stress and thermal influences. To this purpose, we show in Figure 7 the stress field inside the not etched cross section waveguide obtained by means of FEM simulations and induced only by the stress localized in the oxide cap layer. In particular, we have assumed *P_i_* = *50 mW*, *σ_i_* = −*400 MPa* and *T* = *T_ref_* = *293 K*. In addition, the spectral shift has been estimated as a function of the cap stress, as shown in Figure 7. Thus, by increasing the module of the cap stress *σ_i_*, the resonance wavelength shift towards higher values with a slope of −*2.3121* × *10*^−*5*^ *nm/MPa*. In any case, this shift cannot be considered as a detrimental effect. In fact, since the *σ_i_* value depends on the fabrication processing and annealing, it can be considered as known and constant.

**Figure 8.**
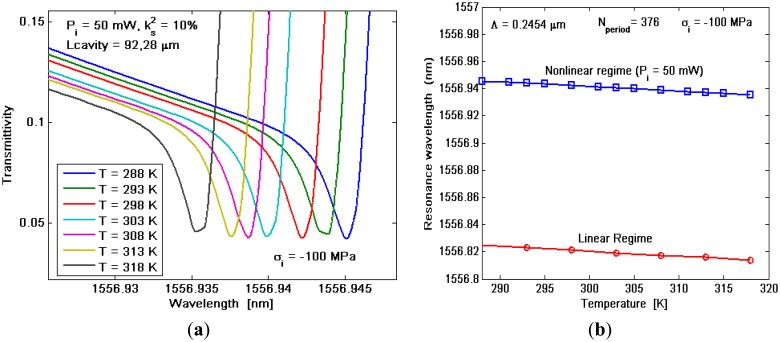
(**a**) Transmittivity *versus* wavelength for different temperatures; (**b**) Resonance wavelength *versus* temperature.

Thus, evaluation of *σ_i_* and curve in Figure 7 lead us to well determine the exact resonance wavelength, since the curve can be used to set the sensor when *σ_i_* is already known. On the contrary, thermal changes introduce not negligible effects as indicated in Figure 8, where the transmittivity spectrum around *λ_2_* is shown for different values of the temperature *T*.

The simulations plotted in Figure 8a,b have been obtained by assuming P_i_ = 50 mW, σ_i_ = −100 MPa, *Λ* ≅ 245.4 nm, and L_cavity_ = 92.28 μm (N_period_ = 376). In particular, Figure 8a indicates that, by increasing the temperature, the transmittivity suffers from a high shift towards higher wavelengths. In fact, by considering ΔT = T − T_ref_ ≠ 0, the thermal effects manifest either a change in the silicon refractive index or, according to Equation (10), a variation in the stress field inside the waveguide cross section with respect to the effect induced by *σ_i_*. In summary, the resonance wavelength shift as a function of the temperature, as in Figure 8b, could assume values sufficiently high to represent a detrimental effect and compromise the sensor sensitivity, as it will be demonstrated in the following.

### 3.2. Sensor Performance

**Figure 9.**
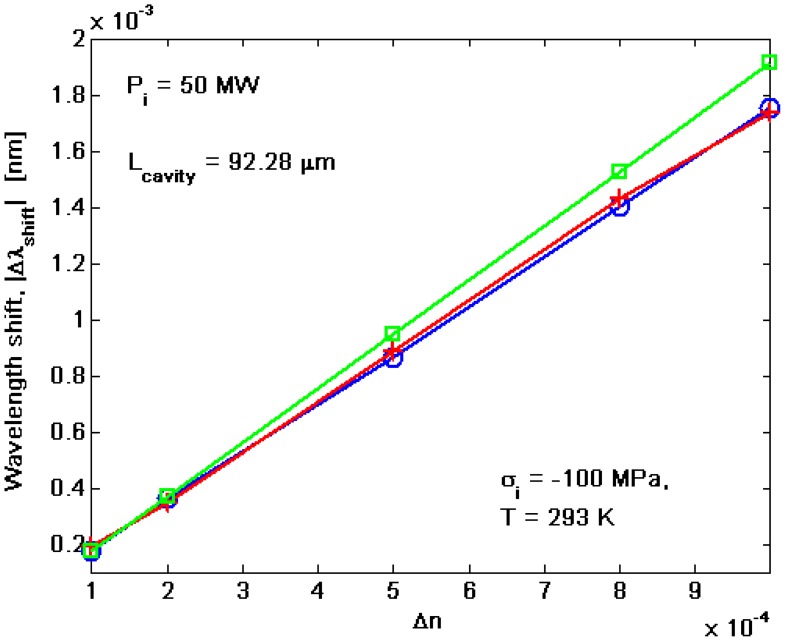
Resonance wavelength shift *versus* the cover refractive index change.

The goal of this sub-section is to analyze the sensor performance in both linear and nonlinear regimes. We have performed a number of parametric simulations according to the integrated and multi-physics algorithm described in previous sections, in order to investigate the performance of the SOI Bragg microcavity resonator. In this sense, by assuming that the cover medium (*i.e.*, water solution) refractive index changes as a function of the concentration of the element to be detected, diluted into the cladding solution, the BMR sensor sensitivity can be written as:

(14) $$S_{r}=\frac{\partial \lambda_{\text{res}}}{\partial n_{\text{cover}}}$$

where *n_cover_* is the refractive index of waveguide cover medium. By considering the results presented previously, the BMR sensitivity depends on the grating and waveguide sensitivity, as well as on the operation temperature. In Figure 9, the wavelength shift as a function of the cover refractive index change *Δn* is shown for different values of coupling factor *κ^2^(T,σ)*.

**Figure 10.**
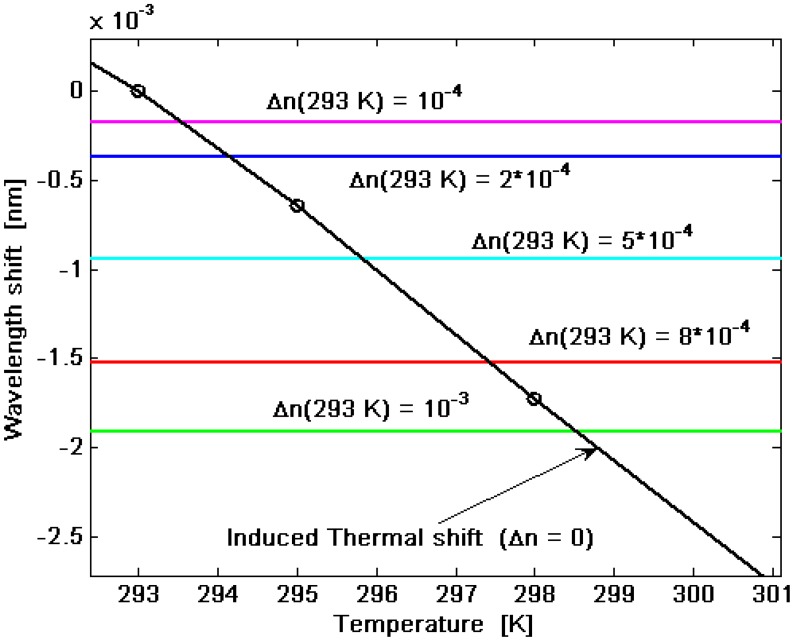
Wavelength shift *versus* temperature. Horizontal lines: wavelength shift for different values of cover refractive index changes.

It is worth outlining that the wavelength shift |*Δλ_shift_*| has been evaluated as the resonance wavelength shifts with respect to the rest condition of the sensor: *Δn* = *0*, and *T* = *T_ref_* = *293 K*. In the simulations, *P_i_* = *50 mW*, *σ*_i_ = − *100 MPa*, *T* =*T_ref_*, Λ ≅ *245.4 nm*, and *L_cavity_* = *92.28 μm* (*N_period_* = *376*) are assumed. The linear profile gives a sensitivity of about 1.7 nm/RIU for each resonant mode of the split doublet. However, as outlined in Figure 8, the temperature changes induce a strong shift of the resonance wavelength. Thus, the sensitivity is limited by the temperature influence, too. Therefore, we can refer to Figure 10 in order to estimate the conditions for a temperature control. The straight line represents the thermal-induced resonance wavelength shift evaluated with respect to the room temperature (*T_ref_* = *293 K*) and *Δn* = *0*. On the contrary, the horizontal lines indicate the resonance wavelength shift, *Δλ_shift_*, calculated at *T* = *T_ref_* = *293K* for different values of *Δn*.

Thus, the intersection points represent the temperature change values with respect to *T_ref_* inducing, in absence of the element in the cladding solution, the same wavelength shift produced by *Δn* value related to the horizontal line intercepted. For example, a temperature change *T* – *T_ref_* = *0.54* °*C* induces the same wavelength shift of *Δn* = *10*^−^*^4^* at *T_ref_*. Consequently, to detect an element concentration change that produces a cladding refractive index change lower than 10^−4^, it is needed to control and keep constant the sensor temperature with great accuracy, within 0.54 °C.

**Figure 11.**
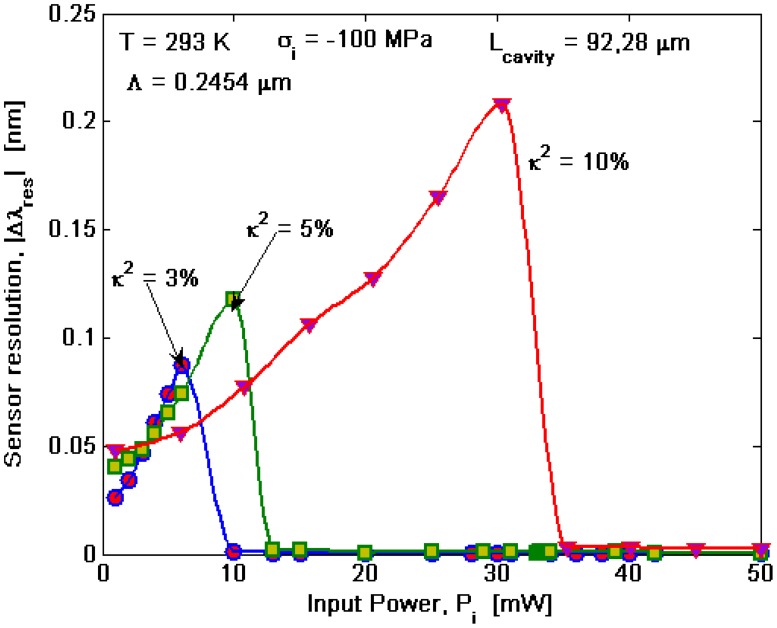
Sensor resolution (detection of level) as a function of input power for different values of the coupling factor between microcavity and external bus waveguide.

Performance of integrated optical sensors based on Bragg microcavity resonator depends not only on *S_r_* but also on their resolution or detection of level (DoL), defined as the minimum detectable change in resonance wavelength. In the Lorentzian resonance shape, this quantity essentially depends on the Full-Width-Half-Maximum *(δλ_FWHM_)* value. In our case, this is true in linear regime or nonlinear regime with *P_i_* < *P̄_ι_*. On the contrary, for *P_i_* ≥ *P̄_ι_* the sensor resolution depends on the separation of very shaped and narrow resonance spikes. It is worth outlining that, under the condition *P_i_* ≥ *P̄_ι_*, we can exploit two advantages with respect to the simple ring resonator configuration, without any Bragg grating. In fact, by designing the BMR in SOI technology, it is possible to reduce the cavity resonance linewidth due to the strong structural dispersion that occurs around the PBG edges [20]. An additional improvement is realized using the nonlinearity-induced spike formation. In Figure 11, the sensor resolution *(|Δλ_res_|)* is shown as a function of the input power for different coupling coefficients. Due to the complexity of the mathematical model proposed, which includes nonlinear, thermal and stress effects, it is not possible to derive a closed-form relationship to calculate |*Δλ_res_|)*, then the curves have been obtained numerically by evaluating the minimum shift acceptable in order to well discriminate the resonance spectrum, following the “Sparrow” criterion. In the simulations, we have assumed *σ_i_* = −*100 MPa*, *T* = *T_ref_*, *Λ* ≅ *245.4 nm*, and *L_cavity_* = *92.28 μm* (*N_period_* = *376*). Consequently, once *κ^2^* has been fixed, |*Δλ_res_*| is shown to increase with increasing the input power up to values where the non linear effects are dominant, giving an abrupt drop of the curve.

Thus, for *P_i_* < *P̄_ι_*, (linear regime) the best sensor performance in terms of resolution can be obtained for lower input powers. In fact, under this condition an increase in power only induces an increase in the losses (TPA and FCA), with consequent spread of the resonance Lorentzian shape. The case with *P* < *P̄_ι_* is very different, giving a strong improvement of |*Δλ_res_*| which is related to the formation of the very narrow spike in the transmittivity spectrum. In addition, the plot shows that, for a given input power, the sensor resolution increases with decreasing *κ^2^*, due to the increment of the microcavity quality factor. Moreover, if the microcavity quality factor increases as well as the enhancement of the resonator, the threshold of nonlinear effects is reduced and, then, the value of *P̄_ι_* decreases by decreasing *κ^2^* (so inducing the abrupt drop of the curves). The main values of sensor resolution (or detection of level) are listed in Table 3 in both operation regimes. It is worth outlining that the curves obtained in Figure 11 under-estimate the resolution, considering it as resolved to Lorentzian profiles (as in our case for *P_i_* < *P̄_ι_*) for a separation equal to *δλ_FWHM_*/3, according to the Sparrow criterion. In this context, by comparing Figure 9 and Table 3, the proposed sensor based on Bragg microcavity resonator can detect cover RI changes as low as *Δn* ≅ 8 × 10^−4^, operating in non-linear regime with *P_i_ ≥ 9.86 mW*, *L_cavity_* = *92.28 μm*, and *κ^2^* = *3%*. However, using the indications proposed in [21] in which two resonance profiles are considered as resolved if they are separated by *δλ_FWHM_*/10 and by appropriately scaling the curves of Figure 11, the proposed sensor can detect a refractive index variation *Δn* ≅ *1* × *10*_−*4*_ RIU in the same operation conditions. This means that the non-linear regime can effectively improve the sensor performance in terms of sensor resolution of at least one order of magnitude (still not using optimal parameters and depending on the coupling coefficient for the given parameters), opening an unexplored approach to RI optical sensing. Moreover, we would underline that, although the obtained DoL results are not to be improved with respect to the best values reported in the literature for RI optical sensors (*i.e.*, *Δn* < *1* × *10*^−^*^6^* [22]), the advantages arising from the split mode structure, consisting in good immunity to the fabrication tolerances and the environmental perturbations, are preserved by operating in a non-linear regime, which is of great importance in RI optical sensing applications.

The proposed approach can be used in spectroscopic differential systems, where the common optical noise of the two resonant split modes can be suppressed.

## 4. Conclusions

In this paper, the physics and the operation of a SOI Bragg grating ring resonator have been investigated as a photonic sensing approach working in both linear and non-linear regimes. The model takes into account all the non-linear effects and optical losses, as well as the thermal and stress influence, too. In linear regime, the device shows a spectral response characterized by a photonic band gap (PBG) behavior. Very close to the band gap edges, it exhibits split resonant modes induced by the grating effect and having a splitting magnitude equal to the PBG spectral extension, almost insensitive to both fabrication tolerances and environmental perturbations. When this device operates in a non-linear regime, exactly in that spectral region showing the split resonant mode structure, our integrated and multi-physics algorithm allows to estimate, in a self-consistent way, the improvement of sensor performance as well as the thermal and stress-induced resonance shift and, then, the sensor temperature control as well. Then, a number of numerical simulations are presented in order to estimate sensor sensitivity and resolution. These simulations confirm that, with respect to the linear regime, the resolution can be improved in a non-linear regime by exploiting the spectral spikes arising from a specific combination of the non-linear effects involved in the sensor architecture. Moreover, the detection of the variation of the distance between the two split modes (*i.e.*, the two spikes generated in non linear regime) allows a spectroscopic differential system to be achieved, in which the common noise, acting on the two split modes, can be easily suppressed. These conclusions will be of great importance in realizing miniaturized linear and non-linear spectroscopic optical sensors, especially in SOI technology platforms, to be used for high performance chemical, biochemical and biological sensing applications.

## Figures and Tables

**Table 1.** Physical parameters used in simulations.

**Parameters**	**Assumed Values**
*E_Y_* (Si)	130 (GPa)
*E_Y_* (SiO_2_)	76.7 (GPa)
*ν* (Si)	0.27
*ν* (SiO_2_)	0.186
*α_therm_* (Si) at 293 K	3.6 × 10^−6^ (K^−1^)
*α_therm_* (SiO_2_) at 293 K	5.4 × 10^−7^ (K^−1^)
*p*_11_ (Si)	−0.101
*p*_12_ (Si)	0.0094
*p*_11_ (SiO_2_)	0.16
*p*_12_ (SiO_2_)	0.27
*α_lass_*	0.5 dB/cm
*τ_eff_*	1 ns
*N_c_*_0_	10^20^ (m^−3^)

**Table 2.** Geometrical parameters used in simulations.

**Parameters**	**Assumed Values**
*Duty cycle*	50%
*h_g_*	50 nm
*H*	450 nm
*H_R_*	150 nm
*H_S_*	300 nm
*W*	700 nm
*h_c_*	350 nm

**Table 3.** Sensor resolution (detection of level).

**Parameters**	**Values**		
Coupling factors (*κ^2^(T,σ)*)	3%	5%	10%
|*Δλ_res_*| at *P_i_* = *1 mW*(Linear regime)	26 pm	40 pm	48 pm
*P̄_ι_*	9.86 mW	13 mW	35.47 mW
|*Δλ_res_*| for *P_i_* ≥ *P̄_ι_* (Non-linear regime)	1.7 pm	2.1 pm	3 pm
